# A Neutralization Epitope in the Hepatitis C Virus E2 Glycoprotein Interacts with Host Entry Factor CD81

**DOI:** 10.1371/journal.pone.0084346

**Published:** 2014-01-06

**Authors:** Zhong Zhao, Lilin Zhong, Elizabeth Elrod, Evi Struble, Li Ma, Hailing Yan, Christine Harman, Lu Deng, Maria Luisa Virata-Theimer, Peter Liu, Harvey Alter, Arash Grakoui, Pei Zhang

**Affiliations:** 1 Division of Hematology, Office of Blood Research and Review, Center for Biologics Evaluation and Research, Food and Drug Administration, Bethesda, Maryland, United States of America; 2 Emory Vaccine Center, Emory University, Atlanta, Georgia, United States of America; 3 Department of Transfusion Medicine, Warren Grant Magnuson Clinical Center, National Institutes of Health, Bethesda, Maryland, United States of America; Saint Louis University, United States of America

## Abstract

The identification of a specific immunogenic candidate that will effectively activate the appropriate pathway for neutralizing antibody production is fundamental for vaccine design. By using a monoclonal antibody (1H8) that neutralizes HCV *in vitro*, we have demonstrated here that 1H8 recognized an epitope mapped between residues A524 and W529 of the E2 protein. We also found that the epitope residues A524, P525, Y527 and W529 were crucial for antibody binding, while the residues T526, Y527 and W529 within the same epitope engaged in the interaction with the host entry factor CD81. Furthermore, we detected “1H8-like” antibodies, defined as those with amino acid-specificity similar to 1H8, in the plasma of patients with chronic HCV infection. The time course study of plasma samples from Patient H, a well-characterized case of post-transfusion hepatitis C, showed that “1H8-like” antibodies could be detected in a sample collected almost two years after the initial infection, thus confirming the immunogenicity of this epitope *in vivo*. The characterization of this neutralization epitope with a function in host entry factor CD81 interaction should enhance our understanding of antibody-mediated neutralization of HCV infections.

## Introduction

Hepatitis C is a major public health problem worldwide. More than 70% of infected people fail to clear the virus during the acute phase of the disease and become chronic carriers [1]. Liver cirrhosis, which develops in about 10 to 20% of chronically infected patients, is linked with a high risk of hepatocellular carcinoma in later life [2]. Hepatitis C virus (HCV)-related end stage liver disease is the leading reason for liver transplantation in the United States [1]. For many years, the standard therapy for hepatitis C consisted of a regimen of pegylated alpha interferon and ribavirin, but showed limited success depending on the viral genotype. This antiviral drug therapy has recently been augmented with the FDA approval of two HCV-specific protease inhibitors, boceprivir and telaprevir. The new drug combination achieved sustained virologic response rates of close to 70% in HCV genotype 1-infected patients in clinical trials [3]. While this new treatment promises a higher cure rate especially for difficult-to-treat HCV genotype 1 infections, the high cost of drug therapy in general still makes this option unaffordable in developing countries. The development of a safe and effective HCV vaccine remains an important goal for the global control of HCV infections. The understanding of antibody-mediated neutralization of HCV infections thus becomes a potentially key step toward the vaccine design.

One of the major challenges in eliciting an effective immunity against HCV is the genetic diversity of the virus [4]–[5]. Overcoming the variability may require the production of specific antibodies that are broadly neutralizing. The HCV envelope glycoprotein E2 has long been considered as an important immunogenic target in efforts to generate a protective immunity. This consideration is largely based on the role of the E2 protein in facilitating the entry of HCV into hepatocytes via interaction, in particular, with the host entry factor CD81 [6]–[8], [27].

In this study, we report that monoclonal antibody (mAb) 1H8 is an HCV-neutralizing antibody which targets the highly conserved peptide sequence APTYSW in the E2 protein from residues 524 to 529. Our study also revealed that this region plays a role in the interaction with host entry factor CD81. Furthermore, our results show that two distinct sets of amino acids within the same epitope can engage in both neutralizing antibody binding and CD81 interaction; this new finding may enhance our understanding of antibody-mediated neutralization of HCV infections.

## Materials and Methods

### Antibodies and plasma samples

Monoclonal antibody 1H8 was produced by Precision Antibody (Columbia, MD) according to its standard procedures. Briefly, BALB/c mice were injected intraperitoneally with recombinant HCV E2 protein (a.a. 384–661) fused to human IgG1-Fc (Invivogen, San Diego, CA). Mice that produced high titers of antibody to the E2 protein were selected for cell fusion to generate hybridomas. The hybrid cells that secreted HCV E2-reactive antibodies were further cloned, expanded, and cultured in BD Cell™ MAb serum-free medium (BD Biosciences, San Jose, CA) at 37°C, 5% CO_2_ for antibody production.

For our study, anonymized patient plasma samples were received from NIH with de-identified number sequences only. None of these samples can be directly or indirectly linked to donor identity. All samples were collected according to protocols approved by the NIH institutional review board. In addition, a separate set of 98 samples was tested in our study. These were taken from the 186 anti-HCV (EIA-1)-positive plasma donations used for generating an anti-HCV immunoglobulin preparation that was previously described [10]–[11]. Each sample was negative for hepatitis B surface antigen, nonreactive for antibody to HIV type 1, and had an alanine aminotransferase level that was less than twice the upper limit of normal. This research study is exempt from IRB and RIHSC review according to 45 CFR 46.101(b)(4).

### Peptide synthesis

By means of standard FastMoc chemistry, all peptides were synthesized on a peptide synthesizer (Model 433A, Applied Biosystems, Foster City, CA) by the Core Laboratory of the Center for Biologics Evaluation and Research, FDA as described previously [12]. Biotinylated peptides were synthesized with a Fmoc-Lys(Biotin-LC)-Wang resin (AnaSpec, San Jose, CA).

### Site-directed mutagenesis and transient expression of recombinant HCV E2 proteins

An HCV E2 construct (E2-16Fc), which spans the region encoding residues 384-661, was made by PCR by using a specific primer pair and then cloned into a mammalian cell expression vector, pFUSE-Fc2-IL2ss (Invivogen). Single site mutations in the E2-16Fc construct were introduced into E2-16Fc according to the QuikChange site-directed mutagenesis kit protocol (Stratagene, La Jolla, CA). The truncated forms of E2-16Fc, i.e., E2-14Fc and E2-12Fc, were likewise constructed by PCR with specific primer pairs to remove the regions encoding residues 571–661 and residues 510–661, respectively, and cloned into the pFUSE-Fc2-IL2ss vector. The constructs were transiently transfected individually into Huh7 cells with Lipofectamine™2000 reagent (Life Technologies, Grand Island, NY). The cell culture media containing the secreted forms of the expressed proteins were collected as supernatants for further analysis.

### Identification of antibody-binding peptides by phage display

The selection of antibody binding peptides from random peptide phage display libraries (New England BioLabs, Beverly, MA) was described previously [13]. Briefly, 10^10^ phages were incubated with a mixture of 1H8 and protein A-coated magnetic beads (Dynabeads® Protein A, Life Technologies) for 20 min at room temperature to allow for sufficient binding. After eight washes with 0.05 M Tris-HCl buffer (pH 7.5) containing 0.15 M NaCl and 0.05% Tween 20 (TBS-T), the bound phages were eluted from the 1H8-protein A complex with 0.2 M glycine solution (pH 2.2). The eluted phages were then amplified in the host strain ER2738. Amplified phages were subjected to two additional rounds of selection with antibody and titrated on LB-agar plates. DNA from single phage plaque was sequenced; the corresponding peptide sequence was then deduced from the DNA sequence. The sequences of the phage-displayed peptide and the HCV E2 protein were then aligned to determine the extent of similarity.

### ELISA

A biotinylated peptide (200 ng) was prepared in 100 µL SuperBlock blocking buffer (Thermo Fisher Scientific, Rockford, IL) and added to each well of a streptavidin-coated 96-well plate (Thermo Fisher Scientific). After incubation at room temperature for 1 h, the plate was washed 4 times with phosphate-buffered saline containing 0.05% Tween-20 (PBS-T) (pH 7.4), followed by incubation with 100 µL of patient plasma (1∶800 dilution) or affinity-purified 1H8 (1:4000 dilution) for 1 h at room temperature. After removal of unbound antibodies by washing 4 times with PBS-T, a goat anti-human peroxidase-conjugated IgG (Sigma–Aldrich, St. Louis, MO) at 1∶3000 dilution in SuperBlock blocking buffer was added to the wells. The plates were washed another 4 times and then kept in the dark for 10 min with 100 µL of TMB Substrate Solution (Thermo Fisher Scientific). The reaction was stopped by adding 50 µL of 1 M H_2_SO_4_. The absorbance (A450 nm) of each well was measured with a Spectramax M2e microtiter plate reader (Molecular Devices, Sunnyvale, CA). A biotinylated peptide (YQPYRVVVLSFELLNAPATV), which was derived from SARS virus [14], was used as the negative control for this assay with 1H8 (1∶4000 dilution); an A450 nm reading of 0.64 (Mean + 2 standard deviations) was obtained, which was used as the cut-off to define the positive.

### SDS-PAGE and Western Blot

Serum-free culture media containing the secreted forms of the E2 protein or E2 mutant were collected 3 days after the transfection of Huh7 cells. The samples were treated with SDS sample loading buffer, heated at 70°C for 5 min, and then loaded into the wells of a 4–12% NuPAGE Bis-Tris precast polyacrylamide gel (Life Technologies). After separation by gel electrophoresis, the proteins were transferred onto a nitrocellulose membrane according to the manufacturer’s instructions. Five percent nonfat dry milk in PBS-T was used to block the membrane for 1 h at room temperature. The blocked membrane was then incubated with 1H8 (1∶1000 dilution), as a primary antibody, for an additional hour. After washing, a horseradish peroxidase-conjugated anti-mouse IgG antibody (1:3000 dilution; KPL, Gaithersburg, MD) was used as the secondary antibody. SuperSignal West Pico chemiluminescent substrate solution (Thermo Scientific) was applied to the membrane according to the manufacturer’s protocol. The data were collected with a FluorChem E Imager (ProteinSimple, Santa Clara, CA).

### Luciferase reporter assay to assess CD81-HCV E2 interaction

The large extracellular domain of human CD81 (residues 113–201) and the E2 protein (residues 384–661) were fused with the gaussia luciferase (New England BioLabs) and the human IgG1 Fc moieties (Invivogen), respectively, to produce the CD81-Luc and E2-16Fc constructs. These constructs were transiently transfected separately into Huh7 cells which later secreted the resulting fusion proteins into the cell culture medium. The amount of protein in the cell culture supernatant was monitored either by Western blot analysis using a specific antibody to the human Fc tag or by measuring the activity of luciferase with the BioLUX® Gaussia Luciferase Flex Assay kit (New England BioLabs). The supernatant (100 µL/well) containing either E2-16Fc or its mutant forms was incubated in 96-well microtiter plates coated with protein A (Thermo Scientific) at room temperature for 1 h. After washing with PBS-T, CD81-Luc supernatant (2.5×10^6^ units of luciferase activity in 30 µL), with 1H8 (10 or 50 µg/reaction) or without 1H8, was added to the wells and incubated for 1 h. After rinsing the plates with PBS-T, a 50 µL mixture of the BioLUX kit substrate and buffer was added to the wells, and the bioluminescent signal intensity was immediately measured with an Infinite F500 microplate reader (TECAN, Männedorf, Switzerland).

### Neutralization Assay

For neutralization assays, the CNS2 Rluc virus was generated by inserting the renilla luciferase gene into the previously described J6/JFH genotype 2a Cp7 virus [15]. 6×10^3^ Huh7.5 cells/well were plated in a 96 well flat bottom plate (Corning, New York). On the next day, 1H8 in 50 µl cell culture supernatant with an antibody concentration of 16.67 µg/mL was serially diluted (1:3) and added to the cell culture, followed by adding 50 µL of CNS2 Rluc virus. After incubation for 72 hours at 37°C, cells were lysed and RLUs were measured with the Promega Renilla Luciferase Assay System (Madison, WI) on a Biotek Clarity Microplate Luminometer (Winooski, VT).

## Results

### Defining the key residues on the HCV E2 protein for the binding of 1H8

**Figure 1:**
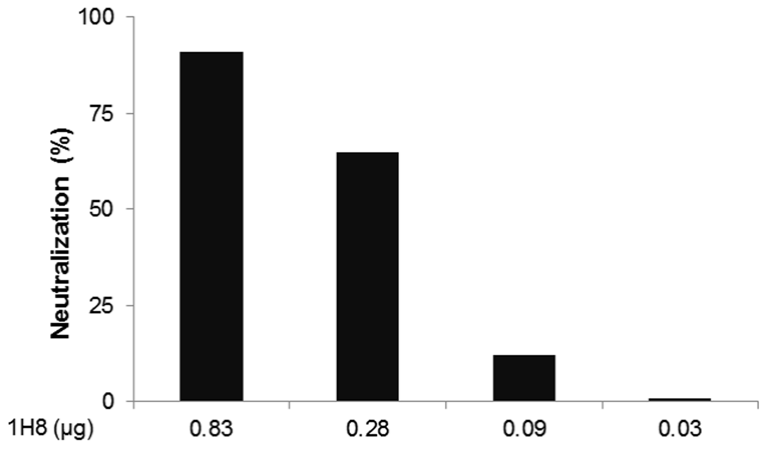
Effect of 1H8 on neutralization of the HCV genotype 2a virus *in vitro*. Hybridoma cell culture supernatant containing approximately 16.67 µg/mL of antibody 1H8 was collected and used in neutralization assay in a 3-fold dilution series [15]. Monoclonal mouse anti-E2 antibody 2C1 supernatant was used as a positive control. Percent neutralization was calculated based on an irrelevant mouse anti-CD4 antibody GK1.5 supernatant [15].

**Figure 2:**
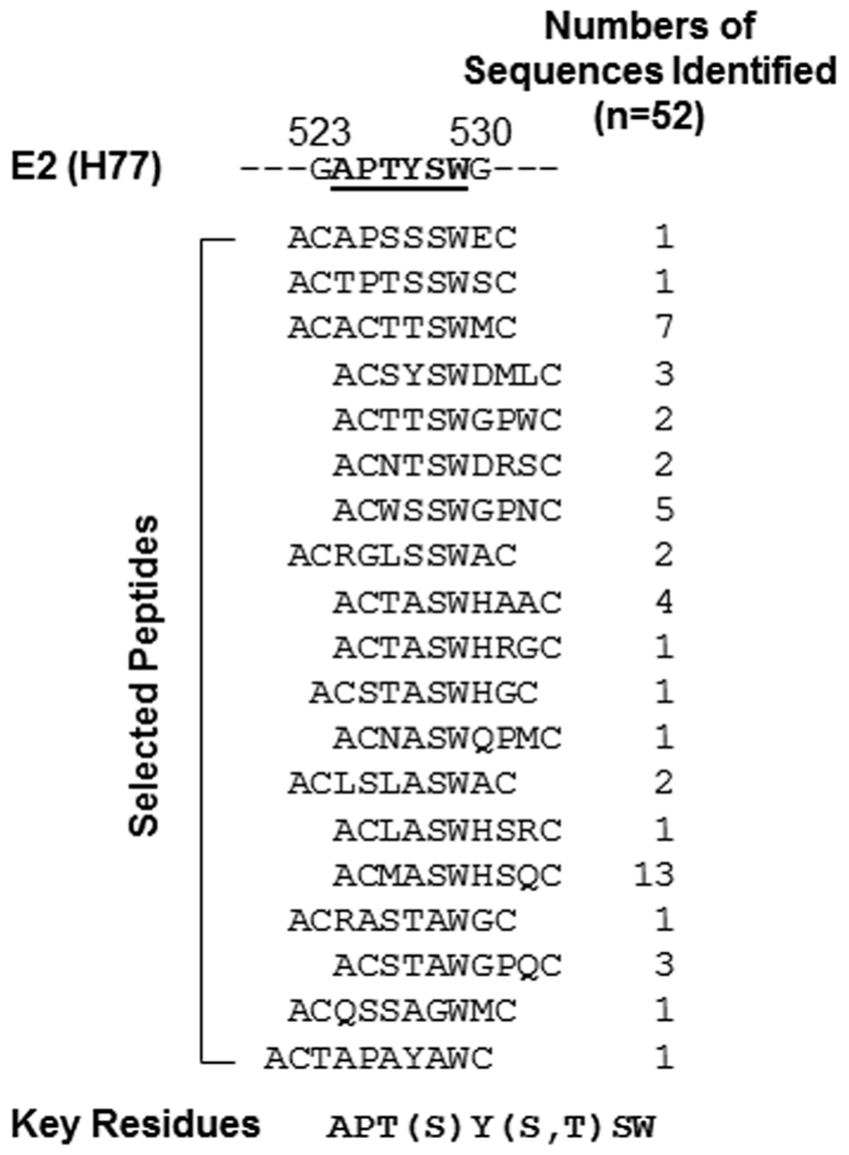
Epitope mapping by screening of random peptide phage display libraries. The amino acid sequences of phage clusters identified after three rounds of screening three random peptide phage display libraries with 1H8. The peptides sequences derived from the phage clones are shown in comparison with a partial linear sequence of the E2, whose sequence is derived from the HCV H77 strain. The key residues for the 1H8 binding are indicated.

We developed a panel of mAbs by immunizing mice with a recombinant HCV E2 protein (residues 384–661 based on the amino acid sequence of the HCV genotype 1a H77 strain), which was fused with a human IgG1 Fc moiety. Among them, mAb 1H8 was found to be a neutralizing antibody because it reduced the infectivity of HCV genotype 2a virus *in vitro* in a dose-dependent manner (Fig 1). The key residues involved in the binding of 1H8 were mapped initially by screening random peptide phage display libraries for 1H8-binding phages (Fig 2). The antibody selected preferentially the phages that displayed peptides containing a W residue in a linear consensus sequence of APT(S)Y(S,T)SW. Based on our analysis of the sequence’s homology to the E2 protein, we predicted that ^524^APTYSW^529^ in the E2 protein was likely to be the binding site of 1H8.

**Figure 3:**
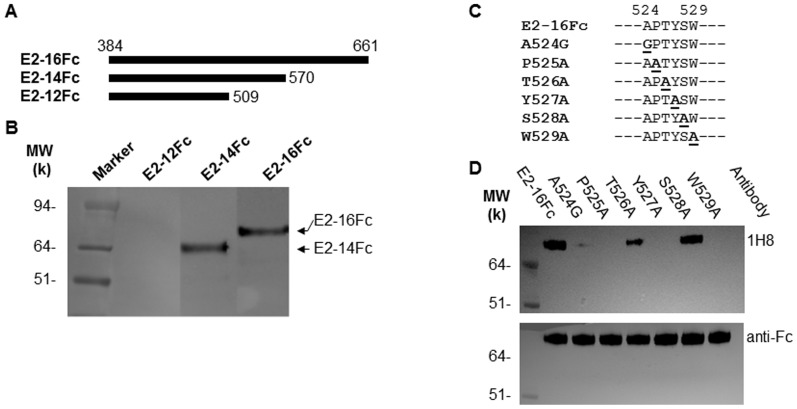
Effect of truncation and mutation of HCV E2 protein on the binding to 1H8. (A) A schematic diagram of the wild-type HCV E2 protein (based on the H77 strain sequence) and its truncated forms that were used in this study. (B) A Western blot analysis was performed to determine the reactivity of mAb 1H8 with the proteins indicated. The secreted forms of these E2 proteins were produced after transient transfection of Huh7 cells. For the Western blots, 1H8 (1∶1000 dilution) and an HRP-conjugated anti-mouse IgG (1∶3000 dilution) were used as the primary and secondary antibodies, respectively. (C) Site-directed mutagenesis of the E2-16Fc construct was performed to determine the key residues for the binding of 1H8. The residues at positions 525, 526, 527, 528 and 529 were replaced by alanine one at a time. At position 524, the alanine was replaced by a glycine. A hyphen indicates an amino acid that is identical to that of the H77 strain sequence. (D) Western blot was performed by using 1H8 (top panel) versus anti-human IgG1 Fc (bottom panel) to determine the effect of the mutations on the binding of 1H8.

To verify further the location of the predicted binding site of 1H8, we generated three C-terminal truncated forms of the E2 protein, which lack the putative transmembrane domain between residues 662–718 (Fig 3A). Our Western blot analysis showed that the E2-16Fc protein (residues 384–661) could be recognized by 1H8. The removal of residues 571–661, as demonstrated with the E2-14Fc protein, did not affect the binding of 1H8. However, a further deletion of the residues between 510 and 570, i.e., as seen with the E2-12Fc protein, resulted in a loss of binding by 1H8 (Fig 3B). These results suggest that the segment between residues 510 and 570, where the sequence of ^524^APTYSW^529^ is located, is involved in the binding of 1H8.

The residue specificity of 1H8 was determined by replacing each of the key contact residues predicted by phage display with an alanine or glycine in the E2-16Fc construct (Fig 3C). A Western blot analysis was then performed to test the specific effect of the substitutions on the binding of 1H8 (Fig 3D). The T526A and S528A mutations did not significantly affect the binding of 1H8, whereas mutations of A524G, P525A, Y527A and W529A reduced the binding. These data were consistent with the prediction from the phage display analysis that residues A524, P525, Y527 and W529 were the direct contact points for the antibody.

**Figure 4:**
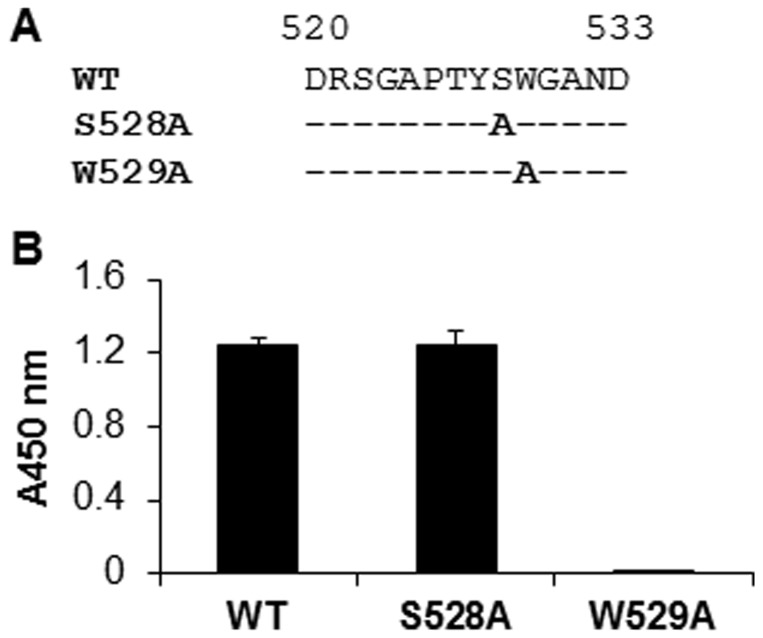
Recognition of a linear peptide from HCV E2 by 1H8. (A) Biotin-conjugated peptides containing amino acids from 520 to 533 of the E2 protein were chemically synthesized to represent the linear epitope of 1H8. Specific substitutions at positions 528 and 529 were introduced (in bold letters), one at the time. A hyphen represents an amino acid that is identical to that of the H77 strain sequence. (B) Detection of the binding of 1H8 to the epitope peptides in an ELISA. A biotin-linked peptide (200 ng) was coated onto a well of 96-well plate which was pre-coated with streptavidin. 1H8, at a 1∶4000 dilution, was used as the primary antibody. HRP-linked anti-mouse antibody was used as the secondary antibody. The data were obtained from at least three independent experiments. The x-axis indicates the peptide used in the ELISA. The y-axis indicates the resulting absorbance at 450 nm, representing the specific binding of 1H8 to the peptide.

We also wanted to know whether the predicted binding site could be recognized by 1H8 in a linear fashion. Several “binding site” peptides containing residues 520–533 were chemically synthesized with or without alanine substitutions at the position S528 or W529 (Fig 4A). The ELISA results showed that the peptide containing the S528A mutation could be recognized by the antibody equally as well as the wild-type peptide. However, the antibody no longer bound the peptide when W529 was replaced by an alanine (Fig 4B). These results confirm that the stretch of residues 520–533 forms a linear epitope for neutralizing antibody 1H8.

### Involvement of the 1H8 binding site in the interaction of E2 with host entry factor CD81

**Figure 5:**
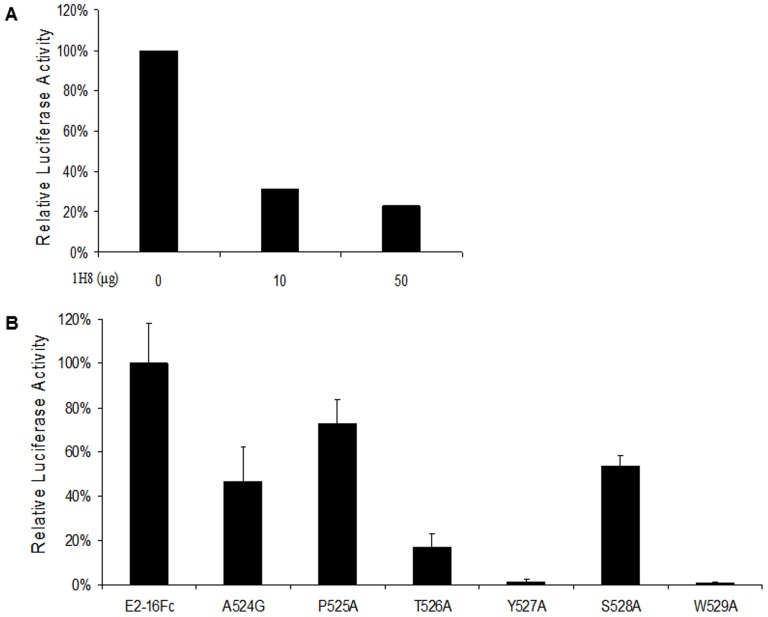
The role of amino acids of the 1H8 binding site in the CD81 interaction of the E2 protein. (A) E2-16Fc was incubated with the Protein A pre-coated 96-well plates at 100 µL/well at room temperature for 1 hour. 30 µL supernatants containing 2.5×10^6^ units of luciferase activity of CD81-Luc was added to each well in the presence or absence of 1H8, and incubated for an additional hour. After an extensive washing, the luciferase activity was measured. The x-axis indicates the 1H8 used in the assay. The y axis indicates the relative luciferase activity, representing the direct interaction between E2-16Fc and CD81-Luc. (B) The interaction of E2-16Fc and its mutants with CD81-Luc was determined under similar experimental conditions as described in (A). The y-axis indicates the relative luciferase activity, representing the direct interaction of E2-16Fc or its mutant with CD81-Luc.

We examined the possibility that the epitope was somehow involved in HCV E2-CD81 interaction, so as to provide an explanation for the neutralizing mechanism of 1H8. We devised a luciferase reporter type of assay with the help of a CD81-Luc recombinant protein to monitor the interactions of the E2 protein with CD81. As expected, the E2-16Fc protein by itself was able to bind to CD81-Luc (Fig 5A). Under the same experimental conditions, we found that the interaction of the E2-16Fc protein with CD81-Luc, represented by the level of luciferase activity, was significantly weakened in the presence of increasing amounts of 1H8 (Fig 5A), thus validating the utility of this luciferase assay.

The blockage of interaction between the E2 protein and CD81 by 1H8 prompted us to determine which residues within the epitope were engaged in the interaction. E2-16Fc protein and its mutated forms were tested for their ability to bind to CD81 in our luciferase assay (Fig 5B). We found that the single site mutations T526A, Y527A or W529A significantly reduced the binding of the E2-16Fc protein to CD81-Luc (i.e., 83.12%, 98.40% and 99.21%, respectively, compared to the wild-type E2-16Fc), thus confirming the observations that were previously reported [27]. Meanwhile, the binding appeared to be less affected by the mutations of A524G, P525A or S528A.

We surveyed the sequence conservation of the amino acid residues 524–529 across HCV strains that were deposited in the Virus Pathogen Database and Analysis Resource (ViPR, http://www.viprbrc.org) (Table 1). The alignment of 1958 HCV E2 protein sequences showed that the four residues, P525, T526, Y527 and W529, are highly conserved. Intriguingly, A524, the residue that we found to be important specifically for 1H8 binding, could be replaced by other amino acids, particularly by valine (948/1958, 48.42%), while maintaining the interface critical for CD81 interaction. This raises the possibility that the binding of 1H8 to the epitope could be modified if the virus contained these substitutions.

**Table 1:** Sequence conservation of amino acid residues 524–529 (n = 1958).

Position	Residue/No. of strains in which it occurred
A524	V 948, A858, T37, I29, L23, N13, D11, H11, M9, X5, E4, Q4, S4, K1, Y1
P525	P1954, A1, R1, S1, X*1
T526	T1947, A4, I1, R1, S4, X*1
Y527	Y1892, F50, H11, C3, S1, X*1
S528	N 838, S531, T408, R85, D39, G19, K11, Y8, A6, X*6, Q2, C1, E1, F1, P1, V1
W529	W1943, F8, R4, C1, G1, X*1

X*: residue not defined; Bold: residues presented in this study

**Figure 6:**
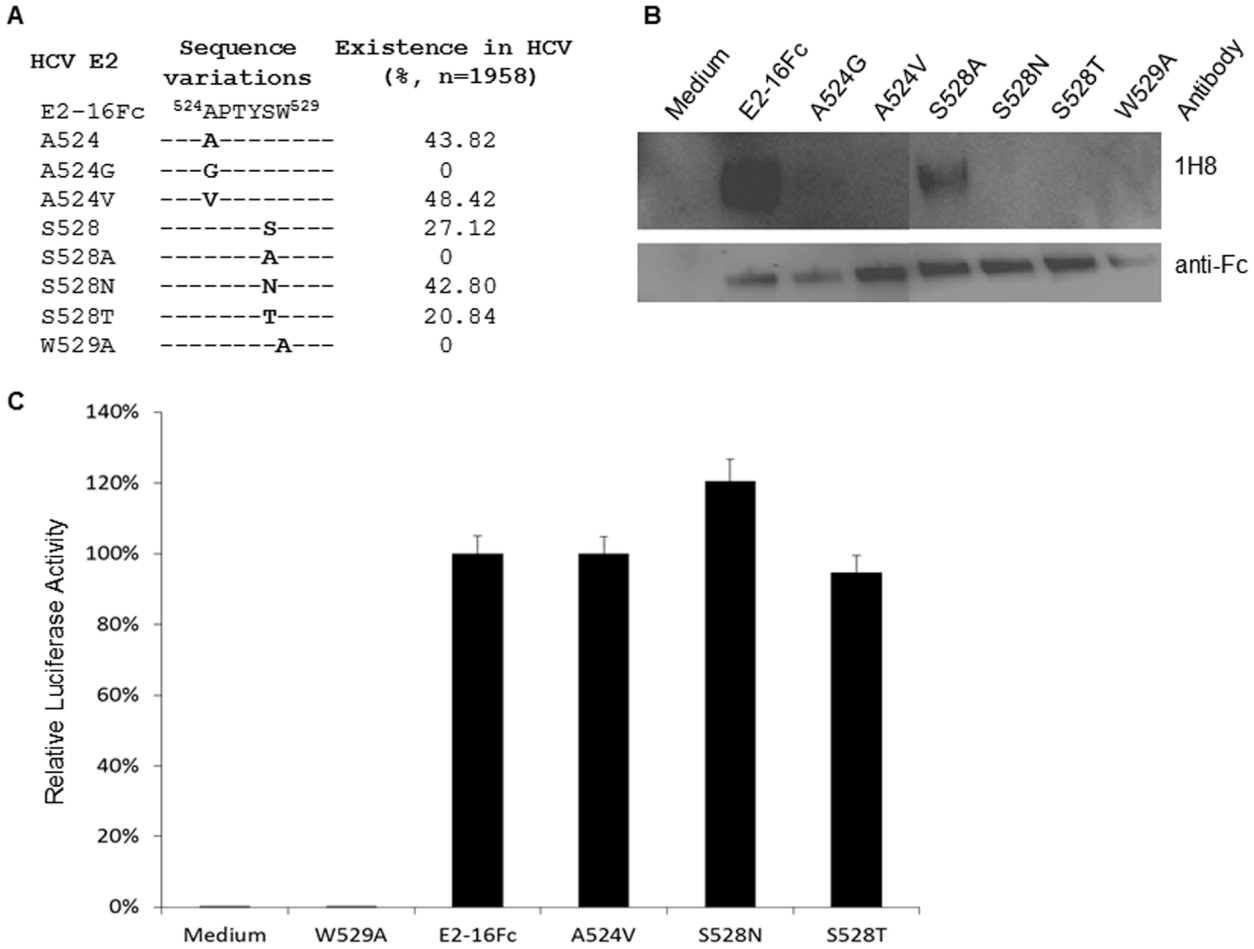
Effect of naturally occurring variants of the E2 protein with specific residue substitutions in the linear epitope on the 1H8 binding and the CD81 interaction. (A) The residues 524–529 derived from the E2 protein of H77 strain, i.e., E2-16Fc, is indicated, which serves as a reference sequence. The bold letter shows the presence of a specific residue at that position. The hyphen indicates any possible amino acids at these positions although the residues at positions of 525, 526,527 and 529 are highly conserved among the strains (Table 1). The prevalence of the strains containing a specific residue (bold letter) in the ViPR database is shown. (B) The reactivity of 1H8 to the E2 protein with an indicated substitution was determined in a Western blot analysis. The secreted forms of these E2 proteins were produced in Huh7 cells. 1H8 (1∶1000 dilution) and an HRP-conjugated anti-mouse IgG (1∶3000 dilution) were used as the primary and secondary antibodies, respectively. The detection with anti-Fc (1∶3000 dilution) was served as a control. (C) Determination of the interaction between the E2-16Fc variants and CD81-Luc in a luciferase-based binding assay. The x-axis indicates the different constructed E2-16Fc used in the assay. The y axis indicates the relative luciferase activity, representing the direct interaction between E2-16Fc and CD81-Luc.

Naturally, amino acid variations, A524V and S528N or S528T, can be found in a high frequency in the HCV strains as deposited in the ViPR database, regardless their genotypes they might be otherwise classified into (Fig 6A). We asked how these variants behave in 1H8 binding or CD81 interaction to the E2 protein. A series of site-directed mutagenesis experiments was carried out to generate two types of variations on the backbone of the E2 protein of the H77 strain (Fig 6A). Our Western blot analysis showed that the substitution of A524 with a valine residue significantly reduced the binding of 1H8 to the E2 protein (Fig 6B). However, as demonstrated by our luciferase-based assay, the interaction of this mutated E2 with CD81 was not affected by the substitution (Fig 6C). This result suggest that while the HCV variants with a valine at position 524 may lose the binding by 1H8, the E2 protein with this A524V substitution, which could be found in 48.42% of strains in the database (n = 1958), may maintain their function to interact with CD81.

We also included the E2 variants with a substitution at position S528 in our experiments. We found that the E2 protein with an S528A substitution did not affect the binding of 1H8, nor did the CD81 binding (Figs 3D, 5B, 6B), suggesting that S528 is not absolutely required for the CD81 binding. Indeed, either S528N or S528T substitutions, which could be seen, respectively, in 42.80% and 20.84% of the HCV strains (n = 1958), did not affect the CD81 binding, although these replacements reduced the binding of 1H8 in the Western blot (Fig 6C). These results suggest that while the binding of 1H8 is residue-specific at the position of 528, the selection of residues at the position 528 is unlikely to be crucial for the E2 protein to interact with CD81.

These observations support the view that at least two sets of amino acids within this short peptide have distinguishable functions, i.e., the recognition by 1H8 or the interaction with CD81 (Table 2). Residues Y527 and W529 were the two residues critical for both antibody and CD81 binding. Residues A524, P525, and S528 (in a residue-specific manner) were critical only for the antibody binding, but not for CD81 interaction. In contrast, T526 was required for CD81 interaction, but not for antibody 1H8 binding. The shared residues, Y527 and W529, for binding of CD81 and antibody likely provided the basis for the neutralizing activity exerted by 1H8 (Fig 1).

**Table 2:** Amino acids linked to antibody or CD81 binding.

Residue	1H8	CD81
A524	+	–
P525	+	–
T526	–	+
Y527	+	+
S528	±	–
W529	+	+

+ Critical; − Not critical; ± Residue-specific.

### Presence of “1H8-like” antibodies in patients with chronic HCV infections

In order to find out whether the binding site of 1H8 was immunogenic enough to elicit epitope-specific antibodies during the course of natural HCV infection, we analyzed plasma samples from 98 patients with chronic HCV infections by an ELISA. The assay cut-off was based on the A450 nm reading of the negative control, a SARS virus peptide. Using A450 >0.64 as the cut-off for the positive, we tested the plasma samples for their reactivity to the wild-type epitope peptide (residues 520–533) and its mutants (S528A and W529A) (Table 3). We found that 53 patient samples (54.08%) reacted positively to the wild-type peptide. Among these 53 wild-type peptide-reactive samples, 35 samples (35.71%) also reacted positively with the mutant S528A. 14 of these 35 samples were negative for W529A. These results demonstrated the existence of “1H8-like” antibodies in patients with HCV infections, thus supporting the notion that the epitope peptide is sufficiently immunogenic in stimulating the host immune system to generate “1H8-like” neutralizing antibodies with a fine residue-specificity.

**Table 3:** Presence of “1H8-like” antibody in patients with chronic HCV infections.

Step 1	Step 2	Step 3
WT (+)	S528A (+)	W529A (–)
54.08% (53/98)	35.71% (35/98)	14.29% (14/98)

Sequential steps for analyzing the data. Plasma samples that gave a positive result in the first step were tested in the second; those that were positive in the second tested in the third.

**Figure 7:**
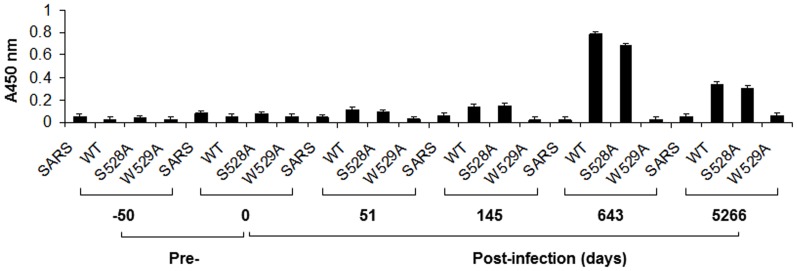
Time course of epitope-specific antibody production in Patient H. Biotin-linked peptides containing the 1H8 binding site and the specific mutation of the binding site were incubated with streptavidin-coated 96-well plates at 200 ng/well. The plasma samples obtained from Patient H were diluted 1∶800 for the ELISA. An unrelated peptide was used as a negative control for the assay. The data were obtained from at least three independent experiments. The x-axis indicates the peptide used in the ELISA and the time when the sample was collected. The y-axis indicates the absorbance at 450 nm, representing the binding activity of the “1H8-like” antibodies in the samples from Patient H.

In order to determine the time course of the production of this particular neutralizing antibody after the initial infection, we tested for the presence of “1H8-like” antibodies in the serial plasma samples of Patient H, a post-transfusion chronic hepatitis C patient whose case has been well-documented [9]. Three peptides, including wild-type, S528A and W529A mutants that were designed to define “1H8-like” specificity of the antibody, were selected for the ELISA. Our results revealed that “1H8-like” antibodies could be detected at day 145 at a relatively low level (Fig 7). Levels of “1H8-like” antibodies had increased significantly by days 643 and remained elevated at day 5266. We concluded that it is possible for hepatitis C patients to produce “1H8-like” neutralizing antibodies, albeit at the chronic stages of HCV infection.

## Discussion

A successful vaccine relies often on its ability to elicit neutralizing antibodies in humans. Paradoxically, during the natural course of HCV infection, neutralizing antibodies are rarely detectable at the early stage of the infection, and if detectable, these antibodies are essentially ineffective *in vivo*, in spite of their neutralizing activity being demonstrated *in vitro* [16]–[17]. As a consequence, patients establish persistent HCV infections even in the presence of appreciable amounts of neutralizing antibodies [9]. Additionally, it has been observed that HCV variants that re-infect the grafted livers of transplant patients are poorly neutralized by antibodies that were present in pre-transplant plasma. In contrast, the viral variants that could no longer be detected following transplantation are efficiently neutralized [18]. These observations support the hypothesis that the profile of antibodies elicited by the virus over the natural course of a patient’s HCV infection, in combination with the high mutation rate of the virus, determines the ultimate pattern of humoral immunity [9]. In the majority of cases the result is that virus-specific neutralizing antibody responses occur too late (or neutralizing antibodies are produced in insufficient amounts) to halt the infection. In line with this thinking, a comprehensive study of clinical samples collected after a single-source outbreak of hepatitis C has shown that neutralizing antibodies produced early in the infection play an important role in the clearance of HCV, thus avoiding chronicity [16].

In the present study, we tested, as a proof of concept, the feasibility of defining an HCV antigenic epitope, which could preferentially elicit neutralizing antibodies against HCV. In addition to showing that residues 524–529 constitute a linear binding site of neutralizing antibody 1H8, we provided the evidence that the same epitope is also involved, through different sets of amino acids, in the interaction of the E2 protein with host entry factor CD81. These results may explain how the 1H8-mediated neutralization of HCV is realized at the interface between the E2 protein and host entry factor CD81.

The genetic diversity of HCV has posed a great challenge to the generation of antibodies capable of neutralizing the various HCV genotypes. With at least six distinct genotypes, HCV exhibits extensive variability, particularly within the envelope genes, which differ by approximately 30% at the nucleotide level [19]–[20]. Even within a single infected individual, HCV may exist as a complex population of genetically distinct variants, termed quasispecies [21]–[22]. Therefore, the conserved residues in the E2 protein that are directly involved in CD81-mediated virus entry would be reasonable targets to elicit neutralizing antibodies. Antibody blocking experiments have indeed shown that several regions of the E2 protein are critical for CD81 binding [6], [23]–[26]. However, because some of the antibodies used in these experiments are conformation-sensitive, it is difficult to confirm whether some of the observed inhibitory effects on the interaction between the E2 protein and CD81 resulted directly from blocking the CD81 binding site of the E2 protein or from antibody-mediated steric hindrance. Despite this difficulty, the majority of the broadly neutralizing conformation-sensitive antibodies reported so far, including those isolated from humans, appear to recognize highly conserved E2 residues, such as W420, Y527, W529, G530, and D535, that are critical for CD81 binding [23]–[24], [27]–[34]. In particular, substitutions at positions 523 and 530 with alanine showed that these two residues, while being a part of the CD81 binding site of the E2 [27], were also important for the neutralization of HCV by several antibodies [32]. Our observation is consistent with previous findings by others who used human monoclonal antibodies and determined that the two conserved residues Y527 and W529 are the antibody contacts of the conformational epitope and are important for the CD81-E2 interaction [27]–[30]. In addition, we further demonstrated that naturally occurring amino acid variations appeared in this linear epitope, such as valine at position 524, and asparagine or threonine at position 528 of the E2 protein may represent a potential mechanism for HCV to escape the 1H8-mediated neutralization. Selectively incorporating certain highly conserved residues, such as Y527 and W529, into this short linear epitope of 1H8, as previously suggested by others and confirmed by us in this study, while taking the variant residues, e.g., V524 and N528 or T528, into consideration for their escape potential, might be worthwhile to explore in order to advance the discovery of an immunogen that will effectively produce neutralizing antibodies against HCV infections.

The ability to dissect the functions exhibited by the residues within the 1H8 binding site gives us the opportunity to monitor the production of “1H8-like” antibodies during the natural course of HCV infection and to correlate their presence with the clinical outcomes. We observed a delayed production of “1H8-like” neutralizing antibody during HCV infection. The weak neutralizing antibody response to E2 protein in chronically infected patients was previously reported by others [9], [35]. At present, we do not know the reason for the delay in “1H8-like” neutralizing antibody production. We speculate that the tertiary structure of viral proteins presented on the surface of the virion, because of the interaction between E1 and E2 as heterodimers, for instance, may prevent the exposure of critical residues necessary for “1H8-like” antibody production in the early phase of infection. Another possibility is that escape variants may be rapidly accumulating in patients at the acute phase of HCV infection which could lead to the failure of any antibody response directed at this epitope. According to our model, some HCV variants, irrelevant to their genotypes, may be able to escape from “1H8-like” antibody-mediated neutralization during the course of natural infections, if the substituted residues in the E2 protein, as changes of alanine to valine at position 524, and serine to asparagine or threonine at position 528, are associated only with antibody recognition without affecting E2-CD81 interaction. We plan to perform a series of experiments in an animal model to systematically investigate if the antibodies elicited after immunization with peptides containing specific substitutions could affect the infectivity of the virus and the breadth of “1H8-like” antibody-mediated neutralization.
